# The healthcare system and client failures contributing to maternal mortality in rural Kenya

**DOI:** 10.1186/s12884-022-05259-w

**Published:** 2022-12-05

**Authors:** Brian Barasa Masaba, Rose Mmusi-Phetoe, Bernard Rono, Damaris Moraa, John K. Moturi, Jane W. Kabo, Samuel Oyugi, Jonathan Taiswa

**Affiliations:** 1 Department of Health Studies, College of Human Sciences, School of Social Sciences, University of South Africa (UNISA), P.O. Box 392, Pretoria, South Africa; 2 Centre of Microbiology Research, Kenya Medical Research Institute (KEMRI), P.O. Box 54840-00200, Nairobi, Kenya; 3 School of Nursing, Kaimosi Friends University, P.O. Box 385, Kaimosi, Kenya; 4 School of Nursing, Kibabii University, P.O. Box 1699, Bungoma, Kenya; 5 Department of Clinical Nursing and Health Informatics, Masinde Muliro University of Science and Technology, P.O. Box 190, Kakamega, Kenya

**Keywords:** Delivery of healthcare, Humans, Kenya, Maternal mortality, Rural population

## Abstract

**Background:**

The global maternal mortality ratio is estimated at 211/100 000 live births in 2017. In Kenya, progress on reducing maternal mortality appears to be slow and persistently higher than the global average, despite efforts by the government’s provision of free maternity services in both private and public facilities in 2013. We aimed to explore and describe the experiences of midwives on maternal deaths that are associated with the healthcare system and client failures in Migori, Kenya.

**Methods:**

An explanatory, qualitative approach method was adopted. In-depth interviews were conducted with the purposively selected midwives working in peripartum units of the three sampled hospitals within Migori County in Kenya. The hospitals included two county referral hospitals and one private referral hospital. Saturation was reached with 37 respondents. NVivo 11 software was used for analysis. Content analysis using a qualitative approach was adopted. Accordingly, the data transcripts were synthesised, coded and organised into thematic domains.

**Results:**

Identified sub-themes: sub-optimal care, staff inadequacy, theatre delays, lack of blood and essential drugs, non-adherence to protocols, staff shortage, inadequate equipment and supplies, unavailable ICU wards, clients’ ANC non-adherence.

**Conclusion:**

In conclusion, the study notes that the healthcare system and client failures are contributing to maternal mortality in the study setting. The major failures are across the pregnancy continuum starting from antenatal care, and intrapartum to post-natal care. This can illustrate that some pregnant mothers are getting sub-optimal care reducing their survival chances. To reduce maternal mortality in Migori County, the key highlighted healthcare system and client failures should be addressed through a multidisciplinary approach mechanism.

## Introduction

The global maternal mortality ratio in 2017 was estimated at 211 deaths per 100,000 live births, a 38% reduction since 2000 when it was 342 per 100 000 live births [1]. Although a decline in trend is noted, most of the mortalities are preventable [1]. Approximately 15% of pregnant women develop some form of obstetric complications during pregnancy and childbirth which is likely to result in maternal death if they fail to receive rapid obstetric interventions [2]. In most developing countries, the major direct causes of maternal morbidity and mortality are hypertensive diseases with eclampsia, postpartum haemorrhage, infections, obstructed labour, ruptured uterus and unsafe abortion [3]. Improving maternal and newborn health is one of the unfinished agendas of the Millennium Development Goals, and it remains a high-priority area in the era of the Sustainable Development Goals [4]. A major goal SDG target 3.1 aims to reduce the global maternal mortality ratio to less than 70 per 100 000 live births by 2030. Despite the United Nations ambition, the WHO estimates that the world will fall short of this target by more than 1 million lives with the current pace of progress [1].

Two regions, Sub-Saharan Africa and South Asia account for 86% of maternal deaths worldwide [1]. Sub-Saharan Africa alone accounts for roughly two-thirds of maternal deaths annually [1]. The region has a lifetime risk of maternal deaths of 1 in 38 with an estimated 530 000 maternal deaths occurring each year [5]. Compared with developed countries, Sub-Saharan Africa cumulates many physical, economic, social, and psychological handicaps, especially in its rural areas: scattered settlements, poor health infrastructure, shortage of qualified healthcare personnel, transportation, and health awareness, and low levels of income and education [6, 7]. A study by Wilunda et al. [8] reported that access to health services in South Sudan is hampered by a poorly functioning health system that is plagued by chronic problems such as a shortage of human resources, lack of health infrastructure and supplies, and weak management [8].

Prior studies on hospital-based maternal mortalities have used a quantitative research approach to present causes and determinants [9–16]. However, only a few have used the current qualitative approach that presents maternal mortality experiences from midwives or healthcare providers. The midwives are key informants that can give more insight into the “background details and circumstances” of maternal deaths beyond the (quantitative) numbers. This later approach has recently been advocated by Stabnick et al. in Ghana [17] and Miskinzod [18] in Tajikistan. In Ghana, the reported system background challenges discussed by providers were a lack of availability of blood products, a high patient volume resulting in an overload of work and poor communication from referral centres [17]. A systematic review in Nigeria, Burkina Faso, Gambia, Guinea, Senegal, and Sierra Leone [19] revealed that 30 facility-level barriers were identified and grouped into six themes (human resources, supply and equipment, referral-related, infrastructure, cost-related, patient-related) [19]. Additionally, Awoonor-Williams and Apanga's [20] mortality study that utilised in-depth interviews with health providers in Ghana showed that lack of logistics, medical, and laboratory equipment, inadequate knowledge about the benefits of antenatal care services as well as non-adherence of healthcare workers to treatment protocols and standard operating procedures were major setbacks to the provision of effective and quality maternal healthcare services in the region [20].

In Kenya, progress in reducing maternal mortality appears to be slow in the past decade from an MMR of 432 in 2010 to 342 in 2017 [21]. This is despite efforts by the government within the last 15 years, such as introducing a reproductive health voucher programme subsidising care in 2006, and provision of free maternity services in public facilities in 2013 [22]. In the present study setting, Migori County is one of 15 counties that account for over 60% of maternal deaths in Kenya. The latest estimate of the county’s maternal mortality ratio (MMR) is 673 deaths per 100 000 live births [23]. Based on this, the researchers aimed to explore and describe the experiences of midwives on maternal deaths that are associated with the healthcare system and client failures in Migori, Kenya.

## Methods

### Study design

An explanatory, qualitative approach method was adopted. In-depth interviews were conducted with the midwives working in peripartum units of the sampled hospitals. The objective covered was to explore and describe the experiences of midwives on maternal deaths that are associated with the healthcare system and client failures in Migori, Kenya. The selection of the approach depended on the research problem and objectives as it allows the benefits of qualitative methods to answer research questions in this study [24].

### Study setting

Healthcare services in Kenya are provided by public health hospitals, private-for-profit facilities and non-governmental organisations. The public health facilities are organised around a four-level system: 1) community services, being the lowest level, 2) primary health services, 3) county referral services, and 4) national referral services. The county has nine county hospitals, 10 private hospitals and 207 primary health centres that include nursing homes and dispensaries. The study was conducted in three sampled hospitals within Migori County in Kenya. The hospitals included two county referral hospitals and one private referral hospital. The researchers used purposive sampling to select three major referral hospitals within Migori County as study sites [25]. Only tertiary referral facilities in Migori County were selected for the study. To maintain ethical anonymity the researchers named the hospitals as AA-County Hospital, BB-Private Hospital and CC-County Hospital.

### Study population

The study population was composed of midwives working within the peripartum units in the sampled hospitals. For this study, a midwife refers to a trained healthcare provider (doctor, nurse or clinician) who is licensed to practice midwifery in Kenya. The midwife needed to be currently working in peripartum units in the selected healthcare facilities in Migori County, Kenya. Lastly, the midwife needed to have experience of more than a year within the department.

#### The sample size for the qualitative study

The study sample size was not predetermined as it was based on the data saturation point principle. Thus, sampling procedure ended when no new major data, themes or codes emerged with each additional interviews or observations [26–28].

#### Calculation of the sample size

The researchers utilised purposive sampling to enrol all midwives working in peripartum units of the three selected hospitals. Accordingly, data saturation of healthcare providers was achieved with 37 participants.

#### Inclusion criteria

##### Midwives

Working in peripartum units in the selected healthcare facilities in Migori County; experience of more than a year within the department.

### Data collection

#### Development and testing of an instrument

The study used a semi-structured interview guide as informed by the relevant literature that has been studied. The researchers used an interview guide to collect data from the midwives presently working in the peripartum units. The midwives’ semi-structured interview guide tool developed in English had open-ended questions on each maternal healthcare determinant the study analysed. They (midwives) were asked about their experiences of recent (within five years) circumstances concerning hospital maternal deaths (clients), scenarios and the associated healthcare system and client factors/gaps.

#### Piloting

A pilot study was conducted at a hospital within Migori County that was not among the three sampled in the main study. The data collection tools (interview guides) were piloted using 10 study participants for each group of participants that were conveniently sampled. An analysis of the pilot study data was done to check the reliability and validity of the data collection tool. No modification of the interview guides was made upon analysing the pilot data collected.

### Data collection process

Data collection unfolded as follows: The researchers had all the needed ethical approvals; they requested the nurse in charge of the maternity department to help them identify the needed study participants. A private room in the peripartum units was requested for the semi-structured interviews at the sampled facilities with the participants who agreed to attend the interviews. Additionally, interview guides were used.

The potential participants were given information about the study and requested to sign consent forms upon voluntarily agreeing to be interviewed. The interview guides developed by the researchers through the use of relevant literature and validated by the supervisor were used to collect data. The researchers used the audio recorder and took notes to ensure that all the data collected is accurate. Participants were assigned unique numbers for anonymity; these numbers were used on the consent form as well. For anonymity and confidentiality, the audio recorder and notes were locked in a locker at a researcher’s home and the consent forms were also locked, but separately from the audio and the notes. These data will be kept safe for five years after the study has been published.

### Data analysis procedure

NVivo 11 software was used, which has the potential to draw rich insights from qualitative data. Content analysis using a qualitative approach was adopted. First, the researchers listened to the audio-recorded data repetitively, checked for completeness, familiarised themselves with it and transcribed it. Accordingly, the data transcripts were synthesised, coded and organised into thematic domains. The researchers identified prominent themes and patterns. Thereafter the themes were categorised and interpretation was made. Direct quotations were also presented to strengthen the research argument.

## Results

### Respondents’ characteristics

The data saturation level was reached with 37 respondents. Most respondents interviewed are working at AA County Hospital (*n* = 20), followed by BB Private Hospital (*n* = 12) and CC County Hospital (*n* = 5). Table 1 shows the qualification of the healthcare professionals interviewed.

**Table 1:** Percentage distribution of professional midwives interviewed (*n* = 37)

Demographic	Category	Percentage (%)
Professional midwives	a. Nurse	54
	b. Midwives	19
	c. Doctor	11
	d. Clinical Officer	16
Years of experience	a.0–3	35
	b.4–7	29
	c. 8–10	18
	d. Above 11	18

As illustrated in Table 1, 54% of the respondents were nurses, 19% midwives, 11% doctors, and finally 16% clinical officers. In addition, 35% of the respondents had less than three years of experience in maternal health, and 29% had four to seven years. Lastly, 18% had eight to 10 years, similar to those with above 11 years of experience.

### Themes and subthemes

The respondents were asked about their experiences of hospital maternal deaths (clients) circumstances and associated healthcare system and client factors/gaps. Saturation was reached with 37 respondents. Accordingly, the researchers developed the following two broad themes: hospital and client-associated failures upon completing the analysis (Table 2).

**Table 2:** Emerging themes and sub-themes

Theme	sub-themes
a) Hospital health care system failures	No communication
	Inadequate skills
	Theatre delays
	Mismanagement
	Lack of blood
	Lack of essential drugs
	Poor quality
	Protocols
	Staff shortage
	Equipments, supplies and consumables, ICU-wards
b) Client related failures	Non adherence to care
	Home delivery
	Failure to recognise danger signs

### Hospital healthcare system-associated failures

#### Poor quality care during antenatal visits

Respondents of this study noted that the majority of the women attend ANC visits during pregnancy. However, vital content information often lacks in their booklets when most are in need – especially antenatal profile data, adequate history and monitoring of vitals, ultrasound and birth plan preparation gaps. Additionally, respondents painfully narrated instances of high-risk clients who default on the required ANC visits with not much contact tracing done. This was illustrated with the following clients:

*A client had undiagnosed multiple pregnancies although she was regularly attending ANC visits, no ultrasound was done; delivery of the first twin was conducted at a peripheral level facility that has no theatre services; she was referred to our hospital; she had C/S for the second twin and died of postpartum haemorrhage-related causes (M1).*

*A client started ANC, however, their ANC profile was not done, missed prophylaxis and mosquito nets defaulted on highly active antiretroviral therapy (HAART); too sad the facility could not follow her up only to be admitted very ill (M2).*

*A client went eight times to the ANC clinic but some important ANC profiles were not done – which entail laboratory tests (M3).*

*A client had ANC monitoring yet the profile was not done (M4).*

### Intrapartum and post-partum care

#### Inadequate resuscitation supplies

From the interview with the respondents, it emerged that hospitals within the study are poorly equipped with emergency resuscitation supplies to manage the most common causes of maternal mortality. It is not uncommon to have stock out of portable oxygen, non-pneumatic anti-shock garments (NASG) and normal saline when critically in need. Illustrating this, respondents in the study shared as follows:

*A client was delivered in the ambulance while on transit from the referring facility before arrival to the referral facility. She was delivered by the midwife who reported the client’s partum haemorrhage development. On reaching the 1*^*st*^* referral facility they did not have blood, the facility offered NASG which they tied to the patient as they went to the 2*^*nd*^* referral facility where the patient was received. Sadly the NASG was removed by the referring person as to return it to the 1*^*st*^* referral facility where it had been borrowed from (M12).*

*Lack of portable oxygen when wheeling a client to theatre (M13)*

*A client had misoprostal prescribed but not administered due to stock out (M14).*

*Lack of essential drugs and normal saline when critically needed (M4).*

#### Referrals

Referral gaps noted in the study by the respondents include communication gaps, inadequate referral notes and delays from the referring facility. Some clients are referred without any referral forms. Also noted is that the peripheral referring facility fails to alert the referral facility of the oncoming client. This indicates that the inappropriate referral reduces the survival chances of the client.

*A client with retained placenta was referred more than three hours after spontaneous vertex delivery (M34).*

*Referral of a client to higher facility was done verbally by the referring facility only for a client to ignore the medical advice and went home, unfortunately she was brought to the referral facility with per vaginal bleeding and diagnosed of ruptured uterus but there was no blood at the referral facility (M34).*

*A client not identified as high risk in post-datism was verbally referred from a peripheral facility. Came in with septic shock that could not be reversed (M16).*

*A facility may refer a client with inadequate information filled on the referral forms (M36).*

*A client referred verbally with no notes given from referring facility* (M32).

#### Ambulance services

Based on the transcripts from the respondents, ambulance services are not available 24 h when needed, especially at the peripheral facilities. This leads to some clients using private vehicles to get to the referral facility. For instance, when a hospital ambulance is taken for routine maintenance, there is no backup plan. Some of the circumstances are as follows:

*A client with retained placenta used private vehicle (probox) as there was no ambulance for transport between facilities (M10).*

*A client missing ambulance services because it had been taken for* maintenance *services to a far City (M11).*

#### Theatre delays

In the study, respondents shared that theatre-related occurrences are contributed by delays due to unavailability of blood, a long theatre list, stock-outs of essential supplies like sutures, water or unavailability of specialist personnel. This can imply gaps in the procurement of essential supplies and maintenance of inventory within the study.

*Lack of sutures and water missing in theatre in our facility is often a reason for referral to another hospital (M25).*

*There was lack of supplies especially IV fluids which delayed transfer of a client to theatre (M26).*

*A client had 48 h delay before she could be taken to theatre because of lack of blood, supplies and long theatre list problems (M27).*

#### Mismanagement/ inappropriate care

In the study, the respondents painfully narrated instances of mismanagement of clients. They highlighted that inadequate care might have reduced the survival chances of the clients significantly. Of note are some quotations, as follows:

*A client was noted to have been admitted of ante-partum haemorrhage unfortunately, Oxytocin drug was administered as part of her care (M5).*

*A client had gauze packing of a cervical tear instead of stitching; she died of post partum haemorrhage (M6)*

*A client was given haematinics with severe anaemia instead of blood transfusion (M7).*

*In-appropriate care as a client was taken to theatre with no blood while anaemic unfortunately- no transfusion during intra-op reduced her survival chances as we lost her (M8).*

*A client who was admitted with sepsis died before appropriate* antibiotics were administered (M19).

#### Protocols and guidelines

Respondents shared some instances of gaps experienced concerning hospital policies and international care protocols required. Of note from the respondents is that peri-mortum C/S is rarely attempted to save the baby as required by the guidelines. The scenarios noted by the study’s respondents are as follows:

*A client had anti-malarials drugs started but not continued only to return in irreversible severe state (M31).*

*Missed to do a peri-mortum caesarean section n(C/S) when the client had cardiac arrest, if peri-mortum C/S could have been done, we could have saved the newborn (M30).*

*A client had investigations ordered (urinalysis, ultra-sound, chest X-ray) but not done (M9).*

*A client started ailing 6 days post delivery was admitted and treated for sepsis and discharged. However 5 days later she is readmitted and never survived (M4).*

#### Patient monitoring and documentation

The respondents in the study highlighted gaps in client monitoring and documentation. Sometimes clients have charts that are inadequately filled or no comprehensive history taken during clerking. Patient monitoring that entails history taking, continuation notes on care and chatting of several tools like input–output, vitals and partographs are essential in care and during change of shift. Examples of narrations were as follows:

*A client delivered at night developed PPH because of the cervical tears that had not been promptly identified, took 2 hours bleeding (lack of patient observation), theatre was delayed due to lack of blood. Later she was indicated for dialysis which she could not get because of renal-dialysis machine breakdown and central venous catheters to be used were out of stock too (M9).*

*Parto-graph inconsistently used in a client- for instance a client reviewed at 2200Hrs by a doctor had a next review at 0600Hrs the following day (M7).*

*There is Input –output charts gaps in documentation- sometimes you find a client on magnesium but no urine and output monitoring chart (M10).*

*A client was not frequently monitored as expected hence the staffs noticed of the post partum haemorrhage (PPH) late when the patient was gasping (M18).*

*An unconscious client admitted was not known to be in labor, until she was in second stage (M22).*

*Documentation gaps ineligible doctor’s notes and inadequate chatting/ history taking on admission/ nursing cardex (M15).*

*A client was on oxygen management had no monitoring of SpO2* (M25).

#### Lack of blood

Respondents in the study strongly indicated inadequate blood in the hospitals. This reduces the survival chances of clients and more so those diagnosed with PPH. Peripheral referring facilities tend to refer clients in the hope of getting blood at the referral hospitals yet most often there is a lack of blood in the whole region. This can indicate the need for blood availability to save mothers. Some unfortunate scenarios are as follows:

*Most often attempts to get blood from nearby facilities are futile and we end up losing a client in need (M6).*

*A client that was HIV reactive was only detected to be anaemic on upon admission and progressed into labor however no blood was available (M7).*

*A client diagnosed of cervical tear, was prepared for theatre but took more than 2 h before being taken because of lack of blood (M8).*

*A client had anemia due to PPH secondary to retained placenta. On successful manual removal there was no blood for transfusion and failed to survive (M9).*

*A client came in with mal-presentation; a C/S was done, she later developed PPH, total abdominal hysterectomy was indicated and performed, Glasgow coma scale was poor. There was no blood and we eventually lost her (M10).*

#### Interdepartmental communication/ teamwork/coordination

Respondents in the study shared instances of lack of coordination as a unit during the management of a client. This can imply that the client is exposed to more unnecessary delays caused by gaps in the team. They highlighted that the peripartum unit works in close coordination with other units like the theatre, laboratory, high dependency unit (HDU), outpatient department (OPD) and radiological centre for the survival of the client. However, sometimes these units have teamwork gaps, as shown:

*A client was weaned off oxygen while waiting to be taken to HDU unfortunately; HDU was not yet ready to receive the admission (M17).*

*Lack of prompt communication between laboratory and maternity departments on stock outs of reagents (M18)*

*Lack of communication of functionality of anaesthetic machine- hence theatre delayed when in need (M23).*

*Nurse covering – did not communicate early to whole team/ departments to anticipate critically ill client arrival (M22).*

*A client had delay of care as the junior healthcare provider delayed* escalation for senior specialist review (M3).

### Hospital capacity

#### Radiological and laboratory equipment/services

As illustrated by the transcriptions from the study’s respondents, there is a persistent lack of radiological services, more so portable scans. Most hospitals within the study lack portable X-ray machines. On the other hand, laboratory services are often out of stock with needed reagents for investigations or machine breakdowns. This can imply that an urgent radiological/laboratory investigation is delayed, hence sub-optimal service delivery to the client. Some illustrations of scenarios are as follows:

*A client who reported at ANC around 30wks gestation at referring hospital later on had rupture of membranes at 38wks and was advised to deliver in a referral hospital (higher). Sadly she delivered at home, went to her former referring hospital for management after 1week. The referring hospital referred her to referral hospital for further management. At the referral hospital liver function tests and urea and electrolytes test- machines had broken down so no investigation done (M8).*

*A client with clinical signs of tuberclosis was admitted in labor and delivered. The client later complicated but no chest X-ray was done as the hospital has no portable X-ray (M1).*

*No portable X-ray, stent services and those clients in need are referred (M36).*

*Not uncommon to have a client die before lab results are out due to delays in care (M29).*

*Shortages of reagents in the lab as multiple clients urea and* electrolytes are not done when needed (M11).

#### Infrastructure

The common hospital incapacity as narrated by the study’s respondents is a lack of intensive care unit (ICU) and inadequate HDU bed capacity for those hospitals that have them. Sadly most often there is low availability of ICU beds in the neighbourhood. Some sadly narrated instances by the respondents are as follows:

*A client prepared for C/S delivered normally, suffered a cervical tear which was repaired, bleeding continued and Explorative surgery was done. We realized uterine rupture and hysterectomy was done. On airway extubation the client could not reverse and she needed ICU services which are unavailable. She succumbed on theatre table. Sadly the patient was breathing spontaneously with no BP readings charted; efforts to get oxygen from maternity ward were unsuccessful hence left on theatre table (M1).*

*A client critically ill but was not referred as it is not easy to get ICU (M34).*

*A client missed antibiotics because of stock out and never got to HDU because beds were full (M17).*

*A client had multi-organ failure, respiratory failure and was on HAART was taken for Emergency CS. She developed secretions making intubation a challenge. Client required HDU but there was no bed (M14).*

*A high risk client not linked to the clinic came in with multiple organ failure at 36wks. However she was taken to HDU a day after surgery because there was no bed available despite saturating at 80% (M13).*

*A client with poly-hydramnios and intra-uterine fetal death was taken to theatre, the condition worsened in theatre. ICU was recommended but no bed was found in the neighbouring facilities. Client remained in theatre as the ICU was being sought though in vain (M9).*

#### Staff shortage

Respondents of the study highlighted that they are at most overwhelmed by the number of admissions with the poorly staffed unit. Additionally, there is a lack of specialized personnel for the most complicated clients in the study. Some scenarios sadly explained were as follows:

*Lack of critical care physician and anesthetologists (M11)*

*Many clients are cared with very few staffs hence poor monitoring of patients (M13).*

*A client delayed to get emergency services in time up to 2 h because no anaesthetist was available at that moment (M15).*

*Clients go undiagnosed because of lack of specialist like physicians and haemoncologists (M17).*

*A client had poorly been monitored because of inadequate staffs, inadequate history taking and documentation (M19).*

*A client is referred after SVD with retained placenta that was conducted by traditional birth attendant at lower level-then complicates- no staffs were available at lower level too (M23).*

*Delay in handling of a client on admission from a referring facility due to staff shortage (M3).*

*Routinely 3 nurses on duty taking care of the whole 65 bed maternity unit at night (M2).*

*No proper history taking and clerking of clients due to shortage staff* (M5).

### Client-associated failures

#### Home deliveries

The study’s respondents highlighted that some women have home deliveries which sometimes cause complications. However, they never promptly seek medical attention upon home delivery. This indicates the high mortality risk associated with home delivery, more so when a complication arises. Some occurrences of clients given by respondents are as follows:

*A client with pre-eclampsia had home delivery worsened and had 4 convulsions, sadly the client came to hospital 9 hours after delivery. Had very high blood pressures on admission and worsening Glasgow coma scale, management was appropriately commenced but never survived few hours later (M37).*

*A client had rupture of membranes, waited for 3 days at home before* coming to hospital, later on developed PPH (M37).

#### Ignorance and failure to recognise danger signs

Respondents in the study highlight that some women are health illiterate and fail to promptly seek healthcare services even with obvious life-threatening signs. Other clients noted in the study that the majority of respondents used herbal drugs instead of modern medicine. This can imply inadequate health education on danger signs among the clients and underestimation of the gravity of the complications [29]. Some of the illustrations of failure to recognise danger signs are as follows:

*A client that had 1-previous scar labored at home for more than 6 h, she came to hospital at 3 cm cervical dilatation (M4).*

*A client is referred to a higher facility but opts to go for a traditional herbalist (M14).*

*A client used herbal medicine believing that the disease is caused by evil spirits yet she had anaemia in pregnancy (M24).*

*A client came in with immune-suppression with hepatic failure. Sadly she had stayed at home for 10 days with PPROM hence missing out on timely care (M2).*

*A client had home abortion, bled close to a month before seeking healthcare service when severely ill to be resuscitated (M9).*

*A client with sepsis convulsed at home and they kept her home for one week while unconscious before seeking healthcare (M7).*

#### Non-adherence

The respondents in the study shared that some pregnant women attend the sub-optimal required number of visits. Unfortunately, some are high-risk clients hence poor monitoring of their progress. This implies that, although the majority of women attend ANC visits in the study, there are adherence gaps. The following are some of the clients highlighted:

*A client who did not attend ANC bled overnight and come the following morning, only to find no blood available (M3).*

*A client came in at term gestation with severe eclampsia but had only attended only one ANC visit in the 1*^*st*^* trimester (M30).*

*A client was not attending ANC despite a positive history of frequent* blood transfusions and hospital contact (M2).

## Discussion

The present study found that various hospital systems and client failures contributed to maternal mortality, as painfully narrated by the respondents (midwives). These gaps were across the pregnancy continuum from antenatal care and intrapartum care to post-partum care. Correspondingly, healthcare providers in the studies by Stabnick et al. [17], Miskinzod [18], Awoonor-Williams and Apanga [20] and Ayanore et al. [30] shared their frustrations with the challenges of the system they encounter, which often result in avoidable maternal deaths [17]. A well-functioning healthcare system should provide effective and efficient care through skilled healthcare providers [31]. The system further requires adequate supplies, equipment, and infrastructure, as well as an efficient system of communication, referral and transport [32] which has been noted as an “enabling environment” [31]. The study’s results highlight sub-optimal ANC care services as some clients had no routine ANC profile, vitals monitoring or ultrasound. Of note is maternal services are free in the study setting. This can imply that access alone is not adequate without improvement in the quality content of service offered at the ANC level. Previously, a study in Kenya noted that while many women receive basic ANC services such as blood pressure monitoring and urine test at least once during pregnancy, many are not receiving these consistently at every visit, as recommended by Kenya’s national guidelines [33]. The situation is even more dire for more advanced services such as ultrasounds, which less than one out of every five women in the sample received, with women who had complications (the group for whom it is recommended) less likely to receive it [33]. Similarly, healthcare staffs in Ghana explained that this might be due to the poorly resourced nature of health facilities in rural communities which tends to make it difficult for them to carry out basic and ordinary medical and laboratory tests as they lack the requisite equipment and logistics [20].

Midwives sorrowfully noted that some clients exhibited poor knowledge of danger signs despite attending ANC visits. ANC is an opportunity to educate women about their health, pregnancy and childbirth, and recognise danger signs [34]. This can also imply that inadequate health education was given to some clients in the present study. In a Somalian study, lack of knowledge about danger signs and warnings during pregnancy and failure to seek prompt treatment from healthcare facilities were the causes of delay in deciding to seek healthcare [35]. Awoonor-Williams and Apanga’s study among healthcare providers deduced that it is paramount for key messages at ANC services and educational campaigns to continuously emphasise the need for birth preparedness plans among pregnant women [20].

Non-adherence was presently noted in some clients that were even high risk. This can illustrate the failure of follow-up within the healthcare system. It is recommended that expectant mothers receive at least eight antenatal visits to check and monitor the health of the mother and foetus [36]. Mothers who attend more ANC visits are also more likely to learn danger signs and to deliver under a skilled healthcare attendant [37].

### Intrapartum and postpartum gaps

Emergency supplies and consumables were demonstrated by the findings to be inadequate. This can indicate that there is low preparedness to handle an emergency client in the current study. Similar to the present findings, a study in Ghana highlighted clients in which moderate anaemia was an indirect cause of maternal mortality [20]. The absence of oxygen and readily available blood at the district hospital exposed how weak rural healthcare services can be [20]. It is most likely that if oxygen and blood were readily available the client could have possibly been saved [20].

Respondents highlight that ambulances in the study are not adequate to meet the patient demand. In support of the findings, a healthcare provider in a Tajikistan study showed that improvement of the referral system among various obstetric health institutions and health providers was a critical factor in reducing MMR [18]. In Malawi, maternal verbal autopsy researchers noted that there were delays in the necessary referrals of deceased mothers from one healthcare facility to another due to the lack of emergency transport [38]. Ensuring that those who develop obstetric emergencies during childbirth are quickly transported to facilities where they can receive quality emergency obstetric care can be the difference between life and death for the pregnant woman and her foetus [39].

Scenarios of inappropriate and mismanagement of clients within the study were painfully noted. This can illustrate negligence and incompetence among healthcare workers to adequately manage clients with life-threatening conditions. Lack of good quality routine care may lead to more or late detection of complications [40]. A substantial body of evidence is emerging that documents low provider skills and limited facility capability to provide good-quality routine and emergency care at birth [41]. Gabrysch and others [41] add that bringing women into a building with a health worker labelled as being skilled is not enough, but rather women should give birth in a health facility with good care that can save lives and prevent ill health [41]. The sad narratives also demonstrate that although the protocols are available, sometimes there is non-adherence to implementation. A study in the Upper-East Region of Ghana among healthcare providers revealed similar maternal healthcare system setbacks [20]. Non-adherence of healthcare workers to treatment protocols and standard operating procedures was found a major setback to the provision of effective and quality maternal healthcare services in the region [20]. Expectedly, a healthcare provider in a Tajikistan study found that adherence to the national standards helped them address the main drivers of maternal mortality and morbidity.

### Hospital capacity

A highly mentioned challenge in the study concerning infrastructure is the lack of an intensive care unit. This implies that patients in critical condition are referred to the neighbouring counties. Unfortunately finding bed spaces in the neighbourhood is a big challenge. In line with the present findings, Miskinzod explored healthcare providers’ perceptions of the situation of maternal mortality and health in Tajikistan [18]. The construction of a new maternity hospital with an intensive care unit (ICU) and a laboratory, which replaced the old maternity department that did not have these structures, was underpinned as a key factor behind the decrease in maternal mortality in Khorog [18]. Infrastructure developments aid in providing emergency life-saving obstetric care immediately without any needless delays and help save women’s lives. A cross-sectional study conducted in South Sudan revealed that the nonexistence of an ICU unit contributed to the higher proportion of women experiencing organ dysfunction or dying due to lack of intensive care [42].

Lastly, the findings showed that the hospitals under study are understaffed. This can imply that care is sub-optimal as there is a high patient-to-staff ratio or a lack of a specialised team. Similar to the present findings, Ayanore et al. examined stakeholder views (healthcare providers) on maternity care shortcomings in three rural districts of northern Ghana [30]. Ayanore et al. explained that some healthcare staff refuse to work in less-resourced areas (mostly rural facilities) accounting for skilled personnel shortfalls in certain rural facilities [30]. A healthcare provider in Tajikistan added that in the past, before providing care (caesarean section) to a client, they had to look for a rheumatologist, an operation nurse and then look for an anaesthetist. However, currently, they have these services/ personnel available, and they can act much faster or can start performing surgeries, hence saving lives [18]. Correspondingly, Ackers, Ackers-Johnson and Ssekitoleko’s [43] study adds that on a typical day in Ugandan public health centres, it is not unusual for no doctors to be present at work [43]. This has led to a total breakdown in referral systems and highly congested referral hospitals where women are treated by intern doctors and students, usually with no supervision [43].

### Strengths and limitations

The study was conducted in hospitals in only one county (Migori) out of the 47 counties in Kenya. It could be difficult to generalise the present findings to other counties. The topic of maternal mortality is regarded as a sensitive issue within the Kenyan healthcare system, particularly by midwives. Some respondents were not freely open due to fear related to the sensitive discussion. By design, a qualitative study does not produce generalisable results beyond the present settings. However, the thematic analysis of experiences from the large sample size of midwives enriched the presented data. They provided clearer demonstrations of hospital and client shortcomings of maternal care experienced in daily practice in a region classified as a hotspot of maternal mortalities in Kenya. Thus stakeholders can utilise the findings provided as a springboard for future quality improvement programmes and studies in the region.

## Conclusion

In conclusion, the study notes that healthcare system and client failures are contributing to maternal mortality in the study setting. These failures are major across the pregnancy continuum starting from antenatal care, and intrapartum to post-natal care. This can illustrate that some pregnant mothers are getting sub-optimal care reducing their survival chances. In addition, inadequate hospital capacities such as understaffing and lack of intensive care units are key problems. To reduce maternal mortality in Migori County, the key highlighted healthcare system and client failures should be addressed through a multi-disciplinary approach mechanism.

## Data Availability

The datasets generated and/or analysed during the current study are not publicly available to protect clients’ and participants’ confidentiality but are available in an anonymised form from the corresponding author on reasonable request.
